# CEP90 Is Required for the Assembly and Centrosomal Accumulation of Centriolar Satellites, Which Is Essential for Primary Cilia Formation

**DOI:** 10.1371/journal.pone.0048196

**Published:** 2012-10-24

**Authors:** Kyeongmi Kim, Kwanwoo Lee, Kunsoo Rhee

**Affiliations:** Department of Biological Sciences, Seoul National University, Seoul, Korea; Cancer Research UK London Research Institute, United Kingdom

## Abstract

Centriolar satellites are PCM-1-positive granules surrounding centrosomes. Proposed functions of the centriolar satellites include protein targeting to the centrosome, as well as communication between the centrosome and surrounding cytoplasm. CEP90 is a centriolar satellite protein that is critical for spindle pole integrity in mitotic cells. In this study, we examined the biological functions of CEP90 in interphase cells. CEP90 physically interacts with PCM-1 at centriolar satellites, and this interaction is essential for centrosomal accumulation of the centriolar satellites and eventually for primary cilia formation. CEP90 is also required for BBS4 loading on centriolar satellites and its localization in primary cilia. Our results imply that the assembly and transport of centriolar satellites are critical steps for primary cilia formation and ciliary protein recruitment.

## Introduction

Centriolar satellites are electron-dense materials concentrated near the centrosome in various cell types [1], [2]. A major biological function of centriolar satellites may be protein targeting to the centrosome and communication between the centrosome and the surrounding cytoplasm [3]. PCM-1 is considered a central scaffold of the centriolar satellites because knockdown of PCM-1 results in the disappearance of the electron-dense satellite structures [4]. PCM-1 depletion also makes other satellite proteins lose their typical localization patterns near centrosomes [5]–[8]. In addition, another group of proteins is responsible for the subcellular distribution of centriolar satellites rather than existence. BBS4, Hook-3, OFD1, FOR20 and Par6α are centriolar satellite proteins whose depletion results in the dispersal of the centriolar satellites [4], [6], [8]–[10]. BBS4 is especially important for transport of centriolar satellites toward centrosomes, because it interacts with p150^glued^, a subunit of the dynein/dynactin motor complex [9]. By contrast, CEP290 is a protein, depletion of which induces abnormal concentric accumulation of PCM-1 granules at the centrosome [5]. Additional members should be discovered to understand subcellular behaviors of centriolar satellites.

Primary cilium functions as a sensor for chemical or mechanical signals from outside the cell [11]. It assembles through an ordered pathway of distinct steps [12]. First, a basal body forms from a mother centriole, migrates to the cell surface and docks onto the actin-rich cortex. Next, the basal body nucleates the outgrowth of axonemal microtubules, which protrude beneath the membrane extension, giving rise to a cilium. A continued supply of ciliary proteins is required for cilia formation. It is believed that centriolar satellites are involved in cilia formation because depletion of selected satellite components results in defects in cilia formation [3].

CEP90 was recently identified as one of centriolar satellite proteins [13]. Depletion of CEP90 caused mitotic arrest, misaligned chromosomes, and spindle pole fragmentation, suggesting a role in spindle pole integrity [13]. In this study, we tested whether CEP90 is critical for centrosome protein recruitment in interphase cells. Our results revealed that CEP90 is required for the centrosomal accumulation of centriolar satellites and eventually for primary cilia formation.

## Results

### CEP90 is required for the accumulation of the PCM-1 granules at the centrosome

**Figure 1:**
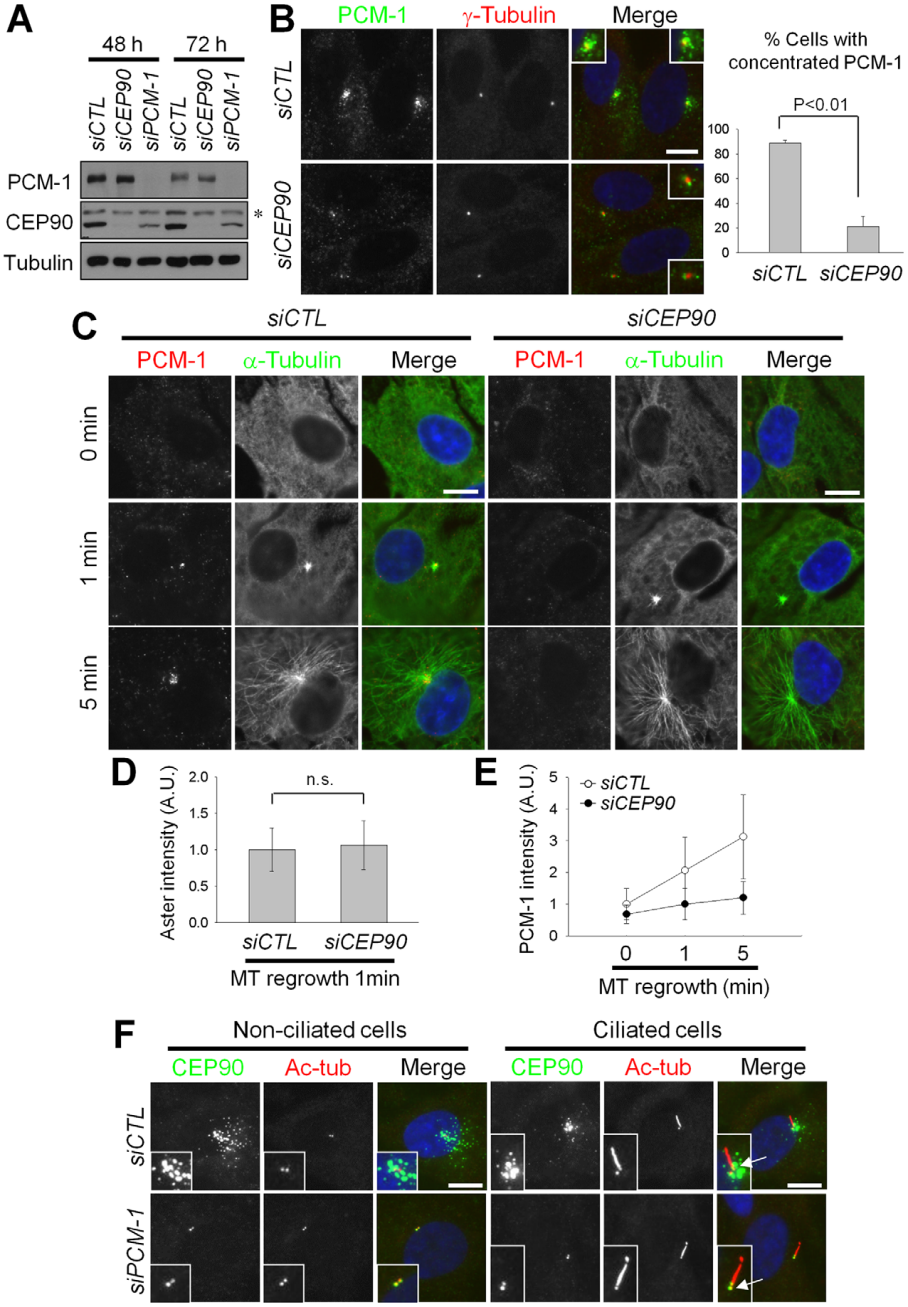
CEP90 is required for the accumulation of PCM-1 granules at the centrosome. (A) RPE-1 cells were transfected with *siCTL*, *siCEP90* or *siPCM-1* and cultured for 48 or 72 h. The cells were subjected to immunoblot analysis with antibodies specific to PCM-1, CEP90 and β-tubulin. An asterisk indicates a non-specific band. (B) The CEP90-depleted RPE-1 cells were co-immunostained with antibodies specific to PCM-1 and γ-tubulin. The resulting images were merged, along with DAPI (nuclear) staining. The insets are magnified views of the centrosomes. The number of cells with centrosome-concentrated PCM-1 was counted. Over 300 cells per experimental group were analyzed in 3 independent experiments. (C–E) The CEP90-depleted RPE-1 cells were cultured in the presence of nocodazole (1 μg/ml) for 3 h and placed in fresh medium for 0, 1 or 5 min. (C) The cells were co-immunostained with the antibodies specific to PCM-1 and α-tubulin. (D) The centrosomal α-tubulin intensities were determined at 1 min in fresh medium. Forty cells per experimental group were analyzed by densitometry in two independent experiments. n.s. indicates not significant. (E) The intensities of centrosomal PCM-1 were determined at the indicated time points. (F) CEP90 localization in PCM-1-depleted RPE-1 cells. The cells were co-immunostained with antibodies specific to CEP90 and acetylated tubulin (Ac-tub). Arrows indicate basal bodies at the base of cilia. The graphs show the mean values and standard errors (B) or standard deviations (D, E). Scale bar, 10 μm.

To gain an insight into the biological functions of the centriolar satellites, we knocked down CEP90 and PCM-1 in RPE-1 cells and observed the resulting phenotypic changes. Transfection of siRNAs specific to CEP90 and PCM-1 effectively depleted the cellular protein levels by 48 h (Figure 1A). PCM-1 granules in interphase cells were concentrated near the centrosome (Figure 1B) [14]. However, the PCM-1 granules in CEP90-depleted cells were scattered throughout the cytoplasm (Figure 1B). The total cellular levels of PCM-1 were not affected by CEP90 depletion (Figure 1A), indicating that CEP90 is required for the centrosomal accumulation of PCM-1.

The microtubule network is critical for the transport of centriolar satellites to the centriolar area [1], [14]. We performed microtubule re-growth assays to determine whether CEP90 is involved with microtubule organization in interphase cells. The results showed that microtubules were assembled from the centrosomes of both control and CEP90-depleted cells as soon as nocodazole was removed from the medium (Figure 1C). The microtubule organization activity of the CEP90-depleted cells was more or less identical to that of the control cells, as determined by the aster intensity of the centrosomes (Figure 1D). The centrosomal PCM-1 levels in control cells were low with nocodazole treatment, but they quickly recovered when the cells were rescued to a nocodazole-free medium (Figure 1C, E). However, recovery of the centrosomal PCM-1 levels in the CEP90-depleted cells was significantly delayed (Figure 1C, E). These results suggest that CEP90 is required for centrosomal targeting of centriolar satellites, irrespective of the microtubule network.

It is known that CEP90 localization at centriolar satellites is also dependent on PCM-1 [13]. CEP90 granules were concentrated near the centrosome with a typical pattern of centriolar satellites in both non-ciliated and ciliated RPE-1 cells (Figure 1F). In PCM-1-depleted cells, the CEP90 signals at centriolar satellites disappeared but those at centrosome remained unaffected (Figure 1F) [13]. This PCM-1 depletion also reduced the cellular CEP90 protein level (Figure 1A). These results are consistent with the proposal, in which PCM-1 functions as a scaffold for the assembly of centriolar satellites [4].

### CEP90 is involved in subcellular distribution of centriolar satellite proteins

**Figure 2:**
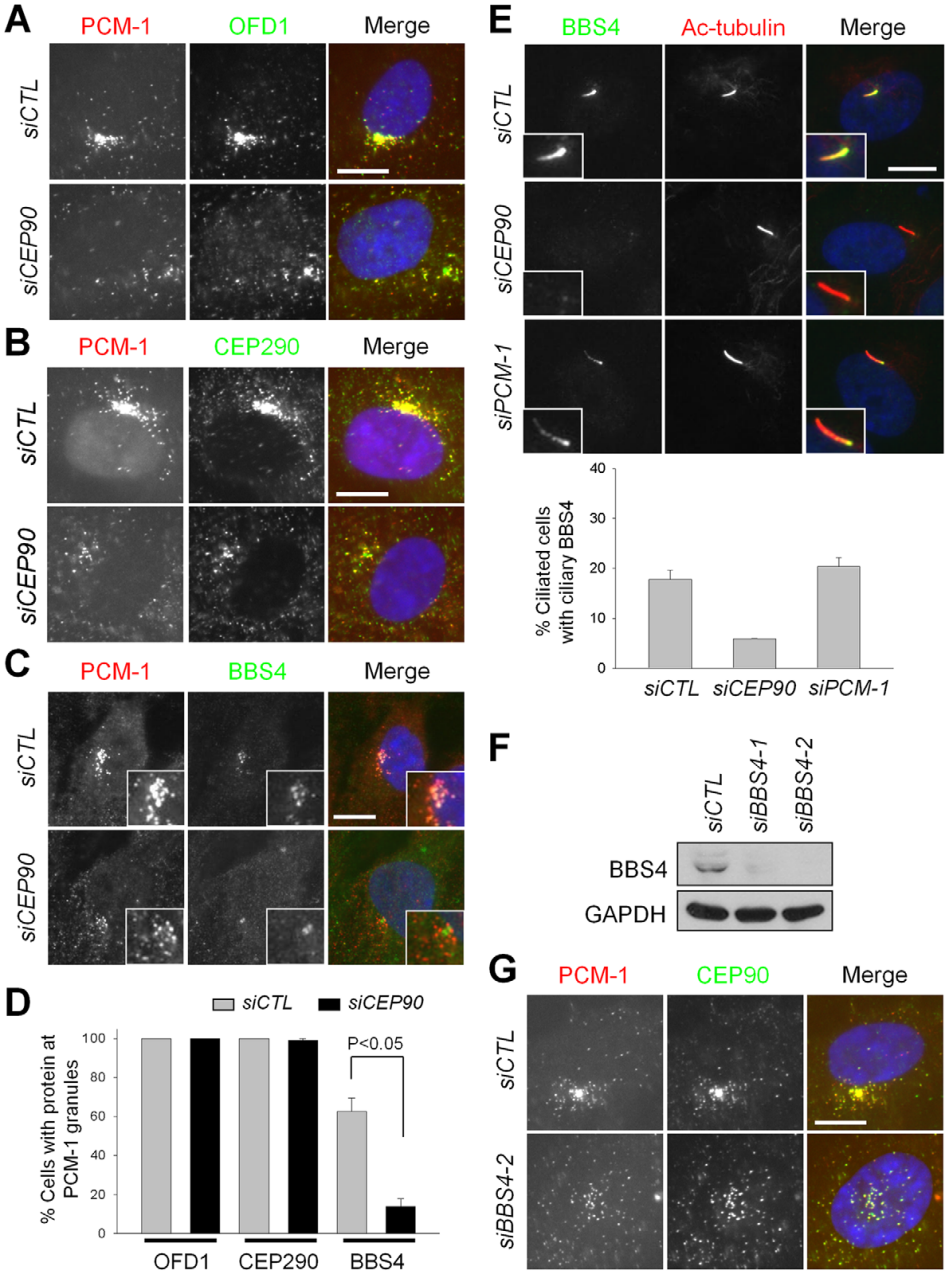
CEP90 is critical for BBS4 localization at centriolar satellites. (A–B) The CEP90-depleted RPE-1 cells were co-immunostained with the antibodies specific to OFD1 (A) or CEP290 (B), along with PCM-1. (C) The cells were co-immunostained for PCM-1 and BBS4. Insets are magnified views of the PCM-1 granules. (D) The number of cells with OFD1, CE290 and BBS4 at the PCM-1-granules was counted. Over 200 cells per experimental group were analyzed in 2 independent experiments. (E) Cilia formation was induced by serum starvation for 24 hours. The cells were immunostained with antibodies specific to BBS4 and acetylated-tubulin. The number of cells with BBS4 at their cilia counted with over 300 ciliated cells in 3 independent experiments. The graphs show the mean values and standard errors (D, E). (F) Depletion of BBS4 with two specific siRNAs in RPE-1 cells. GAPDH is a loading control. (G) PCM-1 and CEP90 were co-immunostained after BBS4 depletion with siBBS4-2. Scale bar, 10 μm.

We also determined whether the other satellite proteins in CEP90-depleted cells are dispersed along with PCM-1. Here, we showed that OFD1 and CEP290 lost a typical granular pattern surrounding the centrosome but were dispersed within the cytoplasm along with PCM-1 in CEP90-depleted cells (Figure 2A, B, D). These results confirm that CEP90 is critical for centrosomal recruitment of centriolar satellites. In contrast, the BBS4 signals in CEP90-depleted cells were hardly detectable at the PCM-1 granules, which were dispersed in the cytoplasm (Figure 2C, D). We examined BBS4 localization at primary cilia after CEP90 depletion. As previously reported, PCM-1 depletion did not change ciliary localization of BBS4 (Figure 2E) [15]. However, CEP90 depletion reduced the number of ciliated cells with BBS4 at their cilia (Figure 2E). In BBS4-depleted cells, both CEP90 and PCM-1 were dispersed in the cytoplasm but remained co-localized at the dispersed granules (Figure 2F, G). These results suggest that CEP90 is required for recruitment of BBS4 and eventual targeting of the centriolar satellites to the centrosome.

### Physical interaction of CEP90 with PCM-1

**Figure 3:**
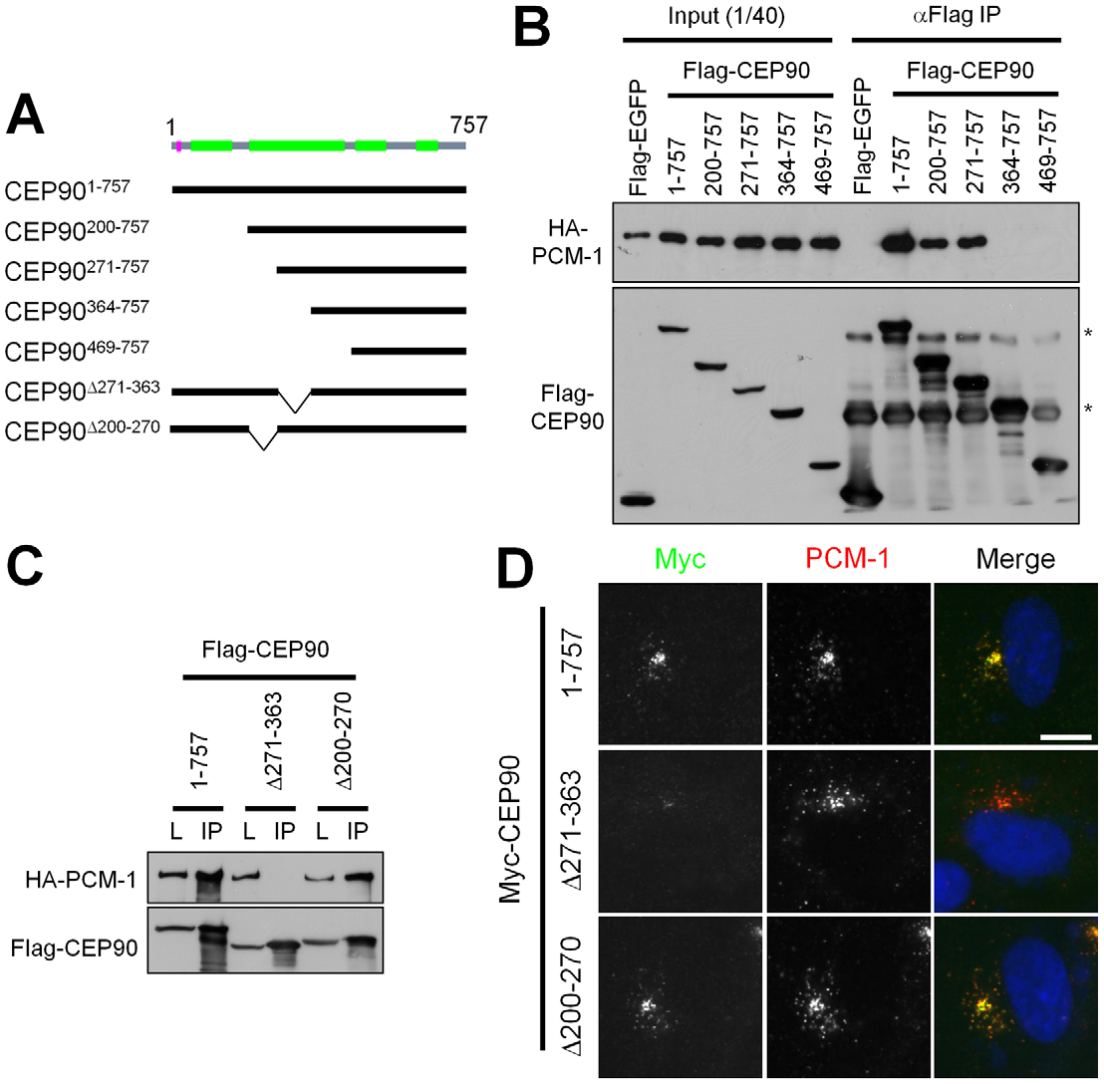
Physical interaction of CEP90 with PCM-1 at the centriolar satellites. (A) Generation of the truncated mutants of CEP90. The green bars indicate the coiled-coil domains of CEP90. (B–C) The Flag-CEP90 and HA-PCM-1 proteins were co-expressed in the 293T cells. The cell lysates (L) were immunoprecipitated (IP) with the Flag antibody followed by immunoblot analysis with the HA and Flag antibodies. Asterisks represent IgG heavy chain or a non-specific band. (D) The Myc-CEP90-expressing RPE-1 cells were immunostained with the antibodies specific to Myc and PCM-1. Scale bar, 10 μm.

We prepared a series of CEP90 truncated mutants and used them to determine an interaction domain with PCM-1 (Figure 3A). Co-immunoprecipitation assays revealed that the 271–363aa residues of CEP90 are responsible for interaction with PCM-1 (Figure 3B). In fact, a CEP90 construct without 271–363 (Flag-CEP90^Δ271–363^) failed to interact with PCM-1 (Figure 3C). Analysis by immunostaining revealed that Flag-CEP90^Δ271–363^ was absent from the centriolar satellites, whereas the control groups such as Flag-CEP90^1–757^ and Flag-CEP90^Δ200–270^ were detected at the centriolar satellites along with PCM-1 (Figure 3D). These results indicate that physical interaction with PCM-1 is critical for CEP90 localization at the centriolar satellites.

**Figure 4:**
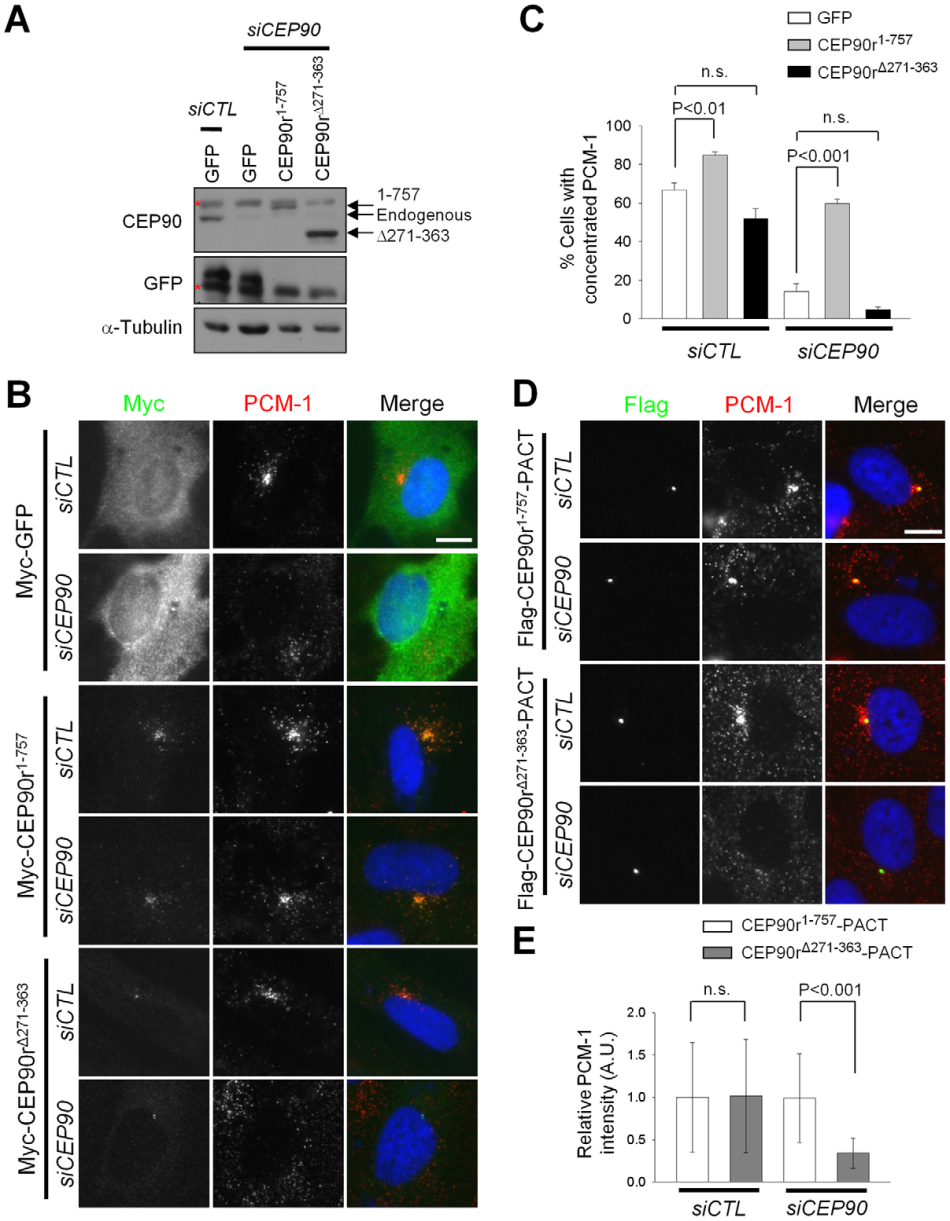
Interaction with CEP90 is critical for centrosomal localization of PCM-1. (A–C) The CEP90-depleted RPE-1 cells were rescued with Myc-GFP or the RNAi-resistant CEP90 constructs (Myc-CEP90^1–757^ and Myc-CEP90^Δ271–363^). (A) Forty-eight hours later, the cells were subjected to immunoblot with the antibodies specific to CEP90 and α-tubulin. Asterisks represent non-specific bands. (B) The cells were co-immunostained with the antibodies specific to Myc and PCM-1. (C) The centrosomal localization of PCM-1 was analyzed in over 200 cells per experimental group. The experiments were repeated 4 times. n.s. indicates not significant. (D–E) The CEP90-depleted RPE-1 cells were rescued with RNAi-resistant CEP90 containing a PACT domain (Flag-CEP90r^1–757^-PACT and Flag-CEP90r^Δ271–363^-PACT). (D) Forty-eight hours later, the cells were co-immunostained with antibodies specific to Flag and PCM-1. (E) The relative intensities of PCM-1 to CEP90-PACT at the centrosome were determined in 20 cells per experimental group. The graphs show the mean values and standard errors (C) or standard deviations (E). Scale bar, 10 μm.

We further examined the importance of CEP90 in subcellular localization of PCM-1 with knockdown-rescue experiments. Immunoblot analysis revealed that endogenous CEP90 was effectively depleted, and ectopic CEP90 proteins resistant to the specific siRNA were expressed (Figure 4A). Subsequent immunostaining analysis showed that the PCM-1 granules were normally accumulated at the centrosome when the CEP90-depleted cells were rescued with the wild-type CEP90 (Myc-CEP90r^1–757^), but not with the CEP90 mutant, which is unable to interact with PCM-1 (Myc-CEP90r^Δ271–363^) (Figure 4B, C). We forced centrosomal localization of the ectopic CEP90 proteins by adding a PACT domain at the C-terminal end and determined subcellular localization of PCM-1 (Figure 4D, E) [16]. The results showed that the PCM-1 failed to localize near the centrosome, even though the Myc-CEP90r^Δ271–363^–PACT protein is concentrated at the centrosome in a similar level with Myc-CEP90r^1–757^-PACT (Figure 4D, E). These results strongly suggest that a physical interaction between CEP90 and PCM-1 is essential for centriolar localization of PCM-1 granules.

### CEP90 interaction with PCM-1 is required for primary cilia formation

**Figure 5:**
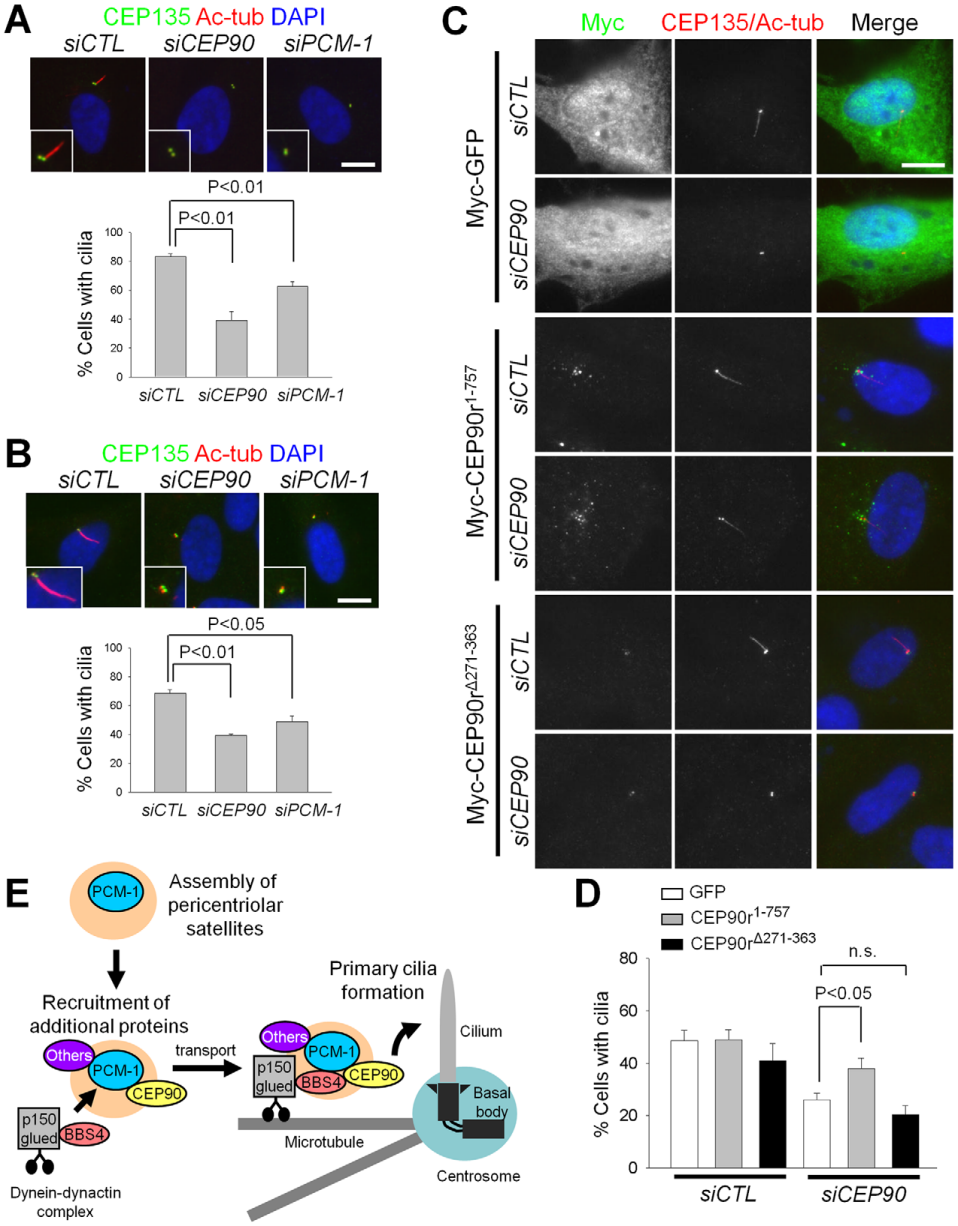
CEP90 is required for primary cilia formation. (A) The CEP90- and PCM-1-depleted RPE-1 cells were cultured in serum-starved medium. The cells were co-immunostained with antibodies specific to CEP135 and acetylated-tubulin. The number of cells with primary cilia (≥1 μm in length) was counted. Over 300 cells were analyzed in 3 independent experiments. (B) The CEP90- and PCM-1-depleted RPE-1 cells were treated with cytochalasin D (100 nM) in serum-starved medium for 16 h. The number of cells with cilia was counted. Over 300 cells were analyzed in 3 independent experiments. (C–D) The CEP90-depleted RPE-1 cells were rescued with RNAi-resistant Myc-CEP90 (Myc-CEP90r^1–757^ and Myc-CEP90r^Δ271–363^). The cells were treated with cytochalasin D (100 nM) in serum-starved medium for 16 h. (C) Basal bodies and cilia were immunostained together with antibodies specific to CEP135 and acetylated-tubulin, respectively. (D) The number of cells with cilia was counted. Over 300 cells were analyzed in 6 independent experiments. The graphs show mean values and standard errors (A, B, D). (E) A model: CEP90 for centrosomal localization of centriolar satellites and primary cilia formation. For assembly of centriolar satellites, PCM-1 functions as a scaffold to recruit CEP90, BBS4 and other components (Others). CEP90 may mediate BBS4 recruiting during centriolar satellite assembly. The centriolar satellites with structural integrity can transport to the centrosome by a microtubule- and dynein/dynactin-dependent manner. In quiescent cells, centriolar satellites near the basal body promote primary cilia formation.

Next, we examined involvement of CEP90 in primary cilia formation. Primary cilia were induced by serum starvation in RPE-1 cells [7], [17]. As reported previously, depletion of PCM-1 reduced the number of ciliated cells (Figure 5A) [5], [7], [17]. At the same time, the primary cilia formation was significantly suppressed in the CEP90-depleted cells (Figure 5A). Ciliogenesis can also be induced with treatment of cytochalasin D, an inhibitor for actin polymerization [18]. Depletion of CEP90 or PCM-1 again inhibited primary cilia formation, which was facilitated with cytochalasin D in serum-free medium (Figure 5B). These results indicate that CEP90 as well as PCM-1 are required for the primary cilia formation in RPE-1 cells.

We performed rescue experiments to confirm importance of CEP90 in primary cilia formation. Cytochalasin D treatment induced primary cilia formation in 50% of the GFP-expressing control RPE-1 cells, and this frequency was reduced significantly in CEP90-depleted cells (Figure 5C, D). The knockdown phenotype was rescued with wild-type CEP90 (Myc-CEP90r^1–757^), but not with Myc-CEP90r^Δ271–363^ (Figure 5C, D). These results suggest that the physical interaction of CEP90 with PCM-1 is critical for primary cilia formation.

## Discussion

In this study, we report that CEP90 physically interacts with PCM-1 at centriolar satellites. The interaction of CEP90 with PCM-1 is important for centrosomal accumulation of the centriolar satellites. Furthermore, this interaction is essential for primary cilia formation. Based on these results, we propose that CEP90 makes centriolar satellites functional for ciliogenesis by recruiting ciliary proteins into the centrosome, where the primary cilium is assembled (Figure 5E).

It is well-known that BBS4 in centriolar satellites links PCM-1 to the dynein motor complex and brings centriolar satellites toward the centrosome [9]. In fact, centriolar satellites in BBS4-depleted cells are dispersed throughout the cytoplasm (Figure 2G) [9]. Here, we reveal that CEP90 is essential for BBS4 loading onto centriolar satellites. The centriolar satellites in CEP90-depleted cells cannot be recruited to the centrosome, possibly because they are absent of BBS4. Although endogenous CEP90 and BBS4 are in the same complex [13], we failed to coimmuoprecipitate the overexpressed proteins (data not shown). This suggests that CEP90 controls BBS4 loading to centriolar satellites in an indirect manner. CEP90 might help to stabilize the interaction between PCM-1 and BBS4. In addition, other satellite proteins, such as Hook-3, OFD1 and FOR20, might also be involved in BBS4 loading to the centriolar satellites.

Recent studies reveal the involvement of centriolar satellites in ciliogenesis. Some ciliopathy proteins, such as BBS4, CEP290 and OFD1, are constituents of centriolar satellites [5], [6], [9], [17]. In addition, PCM-1 and FOR20 have no known linkage with ciliopathy, but are required for ciliogenesis [5], [8], [17]. Our results suggest that CEP90 might function in two steps for cilia formation (Figure 5E). First, CEP90 may be involved in the loading of cilia precursor proteins to centriolar satellites. Second, CEP90 is required for delivering the precursor proteins on centriolar satellites to the vicinity of basal bodies where primary cilia are assembled.

Our results imply that the assembly and transport of centriolar satellites are critical steps for primary cilia formation and ciliary protein recruitment. Detailed mechanisms in the subcellular transport of centriolar satellites should be investigated in the future.

## Materials and Methods

### DNA constructs and Antibodies

The HA-mouse PCM-1 construct was kindly provided by Atsushi Kamiya (Johns Hopkins University School of Medicine, Baltimore, Maryland, USA). CEP90 constructs were made by PCR using CEP90 cDNA as a template [13]. In rescue experiment, RNAi-resistant CEP90 construct (*CEP90r*) was used [13]. For centrosomal targeting of CEP90 protein, 3112–3336 amino acids of pericentrin (PACT domain, GenBank accession number: NM_006031.5) were tagged at the C-terminus of the protein [16]. Anti-CEP90, -BBS4 and -CEP135 rabbit polyclonal antibodies were raised as described previously [13], [19]. Anti-PCM-1 rabbit polyclonal antibody was raised against GST-PCM-1^1409–2024^ and affinity-purified. Anti-ODF1 antibody was kindly provided by Andrew M. Fry (University of Leicester, Lancaster Road, Leicester, UK). We purchased antibodies against CEP290 (ab84870, Abcam), α-tubulin (DM1A, Sigma-Aldrich and B-7, Santa Cruz Biotechnology), β-tubulin (D66, Sigma-Aldrich), acetylated α-tubulin (6-11B-1, Sigma-Aldrich and K40, Cell signaling technology), γ-tubulin (C-20, Santa Cruz Biotechnology), GAPDH (6C5, AMBION), Flag (M2, Sigma-Aldrich), HA (HA-7, Sigma-Aldrich) and Myc (9E10, Covance and 9B11, Cell signaling technology).

### Cell culture and transfection

The hTERT-RPE-1 cells were kindly provided by Dr. Kyung S. Lee (National Institutes of Health, Bethesda, USA) [20]. The hTERT-RPE-1 cells were cultured in DMEM/F12 (1∶1) medium, supplemented with 10% FBS and 293T cells were cultured in DMEM, supplemented with 10% FBS. Cells were grown at 37°C and 5% CO_2_. For RNAi experiments, we used *siCTL* (scrambled sequence for control) (5′-GCA AUC GAA GCU CGG CUA CTT-3′), *siCEP90* (5′-GCA GCU GAC AGA GAC AUA UTT-3′), *siPCM* (5′-UCA GCU UCG UGA UUC UCA GTT-3′) [14] and *siBBS4* (*siBBS4-1*: 5′-GCA CUG ACU UAU GAC CCU ATT-3′ and *siBBS4-2*: 5′-GCU UGU GCU GUU CCA GAA ATT-3′). The siRNAs and plasmid DNAs were transfected into RPE-1 cells using RNAi MAX reagent (Invitrogen) and Fugene HD (Roche), respectively. For immunoprecipitation, CEP90 and PCM-1 constructs were co-transfected into 293T cells by using PEI method [19].

### Primary cilia induction

Twenty four hours after siRNA transfection, hTERT-RPE1 cells were transferred to the medium supplemented with 0.2% FBS and incubated for additional 24 or 48 hours [7]. Or, the cells were incubated with 100 nM Cytochalasin D in the medium supplemented with 0.2% FBS during last 16 hours of 48 hours RNAi [18].

### Immunoprecipitation

For immunoprecipitation, 293T cells were incubated for 30min on ice with lysis buffer (50 mM Tris-HCl at pH 7.5, 150 mM NaCl, 1 mM EDTA, 10 mM NAF and 1% Triton X-100) containing protease inhibitor cocktail (Sigma). The lysates were centrifuged at 12,000 rpm for 20 min at 4°C. To precipitate flag-tagged CEP90 proteins, the supernatants were incubated with anti-Flag M2 affinity gel (Sigma) for 90 minutes at 4°C. The beads were washed 3 times with same lysis buffer and suspended in SDS-PAGE sample buffer for immunoblot analysis.

### Immunocytochemistry, fluorescence microscopy and analyses

The hTERT-RPE-1 cells were cultured on a 12-mm coverslip and fixed with cold methanol for 10 minutes or 3.7% PFA for 15 minutes. For observation of primary cilia, microtubules were depolymerized by cold treatment for 45 minutes, before methanol fixation [17]. Immunofluorescence staining was performed as described before [13]. Immunostained samples were observed by a fluorescence microscope (Olympus IX51) equipped with a CCD (Qicam fast 1394, Qimaging) camera. Image processing and intensity measurement of were performed by using ImagePro 5.0 (Media Cybernetics, Inc.) and Image J (NIH). The intensities of centrosomal PCM-1 and microtubule aster were calculated by subtracting background intensity from the sum intensity of fixed area at centrosome. The graphs were made and statistically analyzed with SigmaPlot (Systat Software, Inc.). P-value was determined by using Student's t-test.
